# MDIntrinsicDimension: Dimensionality-Based Analysis of Collective Motions in Macromolecules from Molecular Dynamics Trajectories

**DOI:** 10.1021/acs.jcim.5c02716

**Published:** 2026-02-26

**Authors:** Irene Cazzaniga, Toni Giorgino

**Affiliations:** Istituto di Biofisica (IBF-CNR), 9327 Consiglio Nazionale delle Ricerche, Milano 20133, Italy

## Abstract

Molecular dynamics (MD) simulations provide atomistic insights into the structure, dynamics, and function of biomolecules by generating time-resolved, high-dimensional trajectories. Analyzing such data benefits from estimating the minimal number of variables required to describe the explored conformational manifold, known as the intrinsic dimension (ID). We present MDIntrinsicDimension, an open-source Python package that estimates ID directly from MD trajectories by combining rotation- and translation-invariant molecular projections with state-of-the-art estimators. The package provides three complementary analysis modes: whole-molecule ID, sliding windows along the sequence, and per-secondary-structure elements. It computes both overall ID (a single summary value) and instantaneous, time-resolved ID that can reveal transitions and heterogeneity over time. We illustrate the approach on fast folding-unfolding trajectories from the DESRES dataset, demonstrating that ID complements conventional geometric descriptors by highlighting spatially localized flexibility, differentiating structural segments, and identifying a metastable configuration.

## Graphical abstract

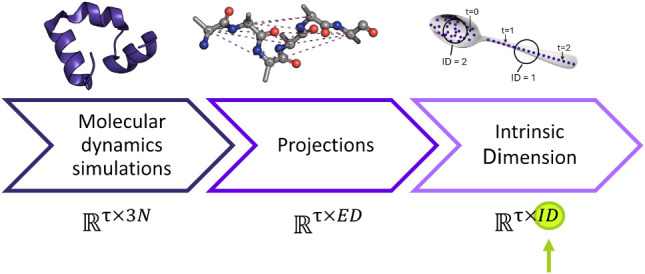

## 1 Introduction

Molecular dynamics (MD) simulation is a computational technique that provides time-resolved, atomistic descriptions of biomolecules by integrating the corresponding equations of motion.^1,2^ The resulting trajectories are high-dimensional and often challenging to interpret directly. While dimensionality reduction techniques embed such data into lower-dimensional spaces^3,4^ an important question is how many variables are minimally required to describe the underlying data manifold, that is, its intrinsic dimension (ID).^5,6^

From a geometric perspective, the ID of a trajectory can be interpreted as the effective number of independent collective coordinates needed to describe the *important* fluctuations actually sampled by the system. In contrast to the formal number of atomic degrees of freedom, which includes many frozen or highly correlated directions, the ID discounts those redundant motions and therefore directly reflects how many modes of motion are relevant on the time scale and in the coordinate representation under study.^7^

Estimating the ID of all-atom trajectories is a challenging and arguably ill-defined task due to several factors. First, the conformational space of biomolecules is inherently high-dimensional, which amplifies data sparsity. Second, meaningful internal degrees of freedom must be distinguished from irrelevant components, such as noise and rigid-body motions. Third, the sampling density is not uniform, both across different regions of conformational space and over time.^8^ The latter is a particularly important consideration for proteins, whose flexibility varies locally and on multiple time scales.^9^

Here, we introduce MDIntrinsicDimension, an open-source Python package that estimates the ID directly from MD trajectories. The package computes internal, rotation- and translation-invariant projections (e.g., backbone dihedrals and inter-residue distances) and applies modern ID estimators provided by the scikit-dimension package.^10^ Three analysis modes are provided, namely whole-molecule, sliding windows along the sequence, and secondary structure elements (Table 1). These complementary views enable both system-level summaries and spatially localized assessments of flexibility.

**Table 1:** Nomenclature on Temporal and Spatial ID Types Adopted in This Package^a^

Locality	Definition	Meaning	Call
**Sequence**	Whole protein	Protein-wide value	intrinsic_dimension()
	Section	ID by overlapping sliding windows	section_id()
	Secondary structure	ID of secondary structure elements	secondary_structure_id()
**Time**	Instantaneous	Time-resolved via local estimators^a^	
	Averaged	Mean of instantaneous ID along a trajectory	
	Overall	ID of the trajectory via global estimators^a^	

a Local estimators can be implicitly converted into global ones and vice versa.

Orthogonally, the package provides three time-resolved representations of ID, namely: *overall*, *instantaneous,* and *averaged*. Overall ID summarizes the dataset with a single value along the trajectory; instantaneous ID is time-resolved, providing a per-frame estimate that can be averaged over the full trajectory or over trailing segments to detect transitions (Table 1). Together, these outputs characterize the complexity of the explored conformational space and its temporal evolution.

We demonstrate our approach on trajectories from the D. E. Shaw Research (DESRES) fast-folding protein trajectory set,^11^ including the Nle/Nle double mutant of the HP35 C-terminal fragment of the villin headpiece (henceforth “villin”) and the N-terminal Domain of Ribosomal Protein L9 (NTL9), and we show that ID complements conventional geometric measures by highlighting heterogeneity across residues and secondary structure elements.

## 2 Methods

The ID calculation workflow comprises three stages: (i) load an MD trajectory; (ii) compute an internal, rigid-body-invariant projection chosen from the available options to obtain a frame-by-feature matrix; (iii) estimate the ID proper using an estimator chosen out of a set of available methods.

### 2.1 Internal Coordinate Projections

Estimating structural properties from molecular dynamics (MD) trajectories benefits from representations that are invariant to rigid-body motions, thereby reflecting only the molecule’s internal degrees of freedom. To this end, each frame of a trajectory is mapped to a vector of *m* descriptors by using suitable projection functions. The resulting projections are stored as an *n* × *m* matrix, where each of the *n* rows corresponds to a trajectory frame and each of the *m* columns to a structural feature.

In practice, we employ two complementary families of descriptors: (i) inter-residue distances or contact counts, typically computed between Cα atoms, which emphasize medium- and long-range couplings within the structure; and (ii) torsional angles, including backbone (ϕ, ψ) and, when relevant, side-chain *(χ)* dihedrals, which capture local conformational variability along the chain.

Trajectory handling and projection calculations are performed using the MoleculeKit library.^12^ Unless otherwise specified, Cα atoms are used for distance calculations, and dihedral angles are expressed in degrees. Periodicity of angular variables can be handled through sine–cosine embedding. Arbitrary projection schemes can also be employed, provided they map each frame to a real-valued feature vector.

### 2.2 Intrinsic Dimension Estimation

To provide an intuition, different classes of methods estimate the dimensionality of the underlying manifold by expressing the same core idea through complementary formalizations. Broadly speaking, distance-based methods look at how the number of neighbors grows as one increases the distance around each point (frame), that is, the power law of how the “volume” of configuration space increases with radius.^13^ Other approaches examine principal axes and how their associated variances decay or related constructions. In all cases, the ID summarizes how many effectively independent directions the system explores and thus plays a role loosely analogous to the number of principal components or time-lagged independent components needed to capture most of the variance in a linear embedding, while being defined in a nonlinear way and allowing for variations in the instantaneous ID.^6^

This package estimates ID using any of the algorithms implemented in scikit-dimension,^10^ which notably include nearest-neighbor, fractal, and likelihood-based operating principles (Supplementary Table S1). We found the Two Nearest Neighbors (*TwoNN*) estimator^8^ to be robust, fast, and well-behaved on MD data, and we adopt it as the default, unless otherwise specified.

Three complementary summary metrics are reported. First, an *overall* estimate, which treats the full set of frames as a single point cloud and yields a single ID value; to mitigate the influence of possibly nonequilibrated initial segments, we additionally report an overall estimate computed on the final portion of the trajectory (whose length can be specified as a parameter). Second, an *instantaneous* estimate produces a time series by evaluating ID in neighborhoods centered at each frame. Third, we summarize this instantaneous series by its mean over the full trajectory and its final portion (*averaged*). In summary, the *instantaneous* ID consists of a vector with as many elements as there are frames in the trajectory, while *averaged* and *overall* ID are real numbers characterizing the whole trajectory. *Averaged* and *overall* ID, while conceptually distinct, were found to yield similar values in our tests (Supplementary Figure S1).

### 2.3 Localizing ID along Sequence and Structure

The package enables space-localized structural analyses to uncover locality and heterogeneity in conformational complexity. First, a sliding-window scheme partitions the protein sequence into overlapping windows of fixed length and stride; for each window, we recompute the internal coordinate projection restricted to the atoms in that window, followed by ID estimation. This produces a profile of ID along the primary sequence, highlighting different types of large-scale coordinated flexibility.

Because neighboring windows share most of their residues, the corresponding ID estimates are strongly correlated. They are best interpreted as a smooth mesoscale profile along the sequence, with an effective spatial resolution set by the window length (and, to a lesser extent, the stride) rather than as independent per-residue measurements. In short, the window should be large enough that the dimensionality of the original embedding space is much larger than the ID, yet small enough to retain local information; this trade-off will be demonstrated in detail with reference to villin trajectories in Section 3.1.5 and Supplementary Figures S2 and S3 (refer to the corresponding captions). On the other hand, increasing the stride has no effect on the overall trends but can speed up the analysis by omitting some windows.

Alternatively, for a structure-based scheme, the package can assign secondary structure using DSSP^14,15^ grouping consecutive residues sharing the same assignment into segments. For each segment, projections and ID values are computed as above. We employ the simplified coil (C), strand (E), and helix (H) DSSP scheme unless stated otherwise; results with the full DSSP alphabet are consistent.

The analysis returns, for each window or secondary structure element, either the overall summary as defined above or the instantaneous series and the corresponding averaged summary, enabling direct comparisons across sequence positions and structural types.

## 3 Results

### 3.1 Case Study: Villin Headpiece

We demonstrate the features of our package using trajectories of spontaneous folding-unfolding transitions from the dataset of fast-folding proteins provided by DESRES in ref 11. We selected the HP35 chicken villin headpiece (PDB: 2F4K), a 35-residue protein with two point mutations to norleucine, K65 (NLE) and K70 (NLE), which increase the folding rate up to 5-fold compared to the wild type (Figure 1). The original trajectory was evaluated using RMSD relative to the folded structure (reference frame 10,400). Based on this analysis, we selected six trajectory segments of 200 ns each (2,000 frames), representing the protein in either folded or unfolded states. These segments are labeled as f0, f1, f2 for the folded state and u0, u1, u2 for the unfolded state, as per Supplementary Table S2.

**Figure 1:**
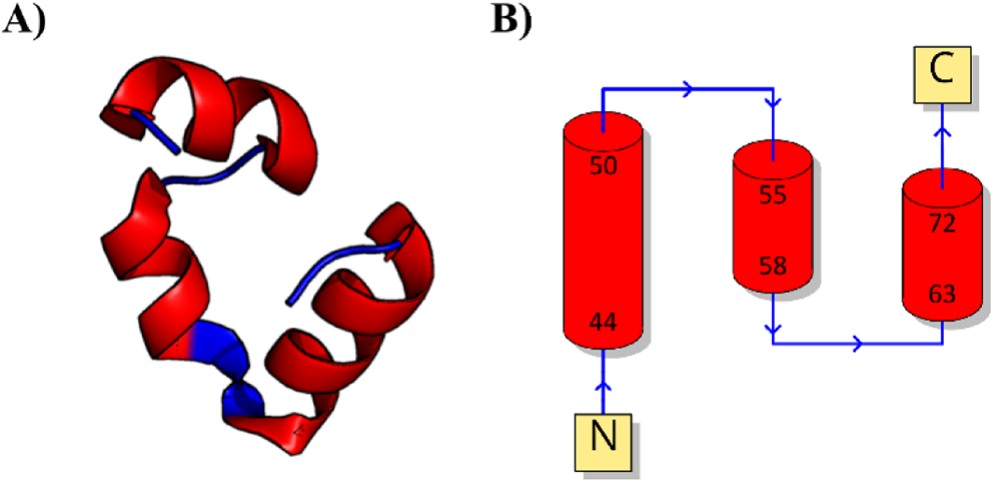
(A) Structure of the HP35 villin headpiece (PDB entry 2F4K) used in the case study and (B) topology diagram.

#### 3.1.1 Estimator Performance

We first evaluated all the estimators provided by scikit-dimension^10^ to assess their ability to handle the complexity of data derived from MD trajectories. Three main aspects were taken into account: (i) the capability to clearly differentiate between the two states (i.e., folded and unfolded); (ii) the ability to distinguish real degrees of freedom from noise; (iii) computation efficiency.

All estimators were able to compute the ID on the protein models used in this article (Figure 2). Most of the remaining estimators succeed in discriminating between folded and unfolded states, generally reporting higher ID values for the folded case. The absolute ID values vary considerably across estimators, showing different discrimination effectiveness between the two states; for example, *MOM* reported very similar values for the two states, while *lPCA* consistently reported the highest ID values measured from the unfolded trajectories (ID > 35).

**Figure 2:**
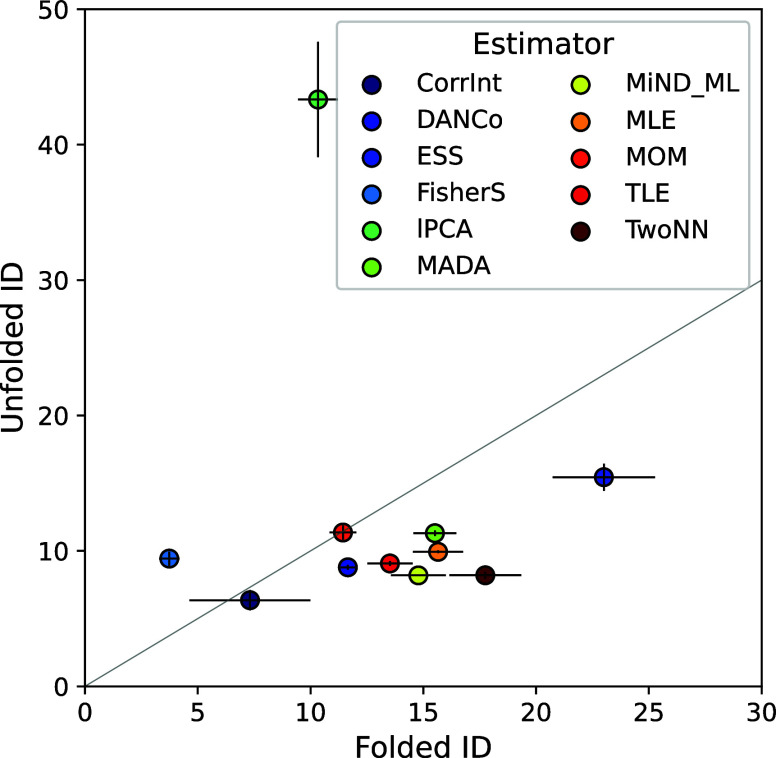
Folded-vs-unfolded ID of the villin dynamic manifold, computed by the estimators available in the scikit-dimension package. Each point represents the mean value of the folded states (*x*-axis) and unfolded states (*y*-axis); error bars indicate standard deviations over three replicas. Methods abbreviations are in Supplementary Table S1. Projection: Ramachandran angles ϕ and ψ, whole protein. *KNN* is not shown due to its high variance.

Based on comparisons across different projections, conditions, and models, we selected *TwoNN* as the default estimator in our functions, as it offers the best compromise between accuracy and computational cost.

#### 3.1.2 Projections

For the sake of illustration, we focus on two main classes of projections, namely, carbon–carbon distances (computed either on Cα or Cβ atoms) and torsional angles. For the latter, we distinguished between backbone dihedrals (ϕ, ψ), which primarily describe backbone flexibility, and side-chain dihedrals (χ), which probe side-chain conformational variability. Both types of torsion were considered in their raw angular form, as well as in their sine/cosine embeddings to account for periodicity.

All of the selected projections are capable of distinguishing between folded and unfolded states (Figure 3). However, the ID values obtained, as well as the gap between the two states, can differ substantially depending on the projection. Remarkably, however, in the case of χ dihedral angles, the trend is inverted: the unfolded state is associated with a higher ID, in contrast to the other projection types, where the folded state typically shows the higher ID. We attribute this inversion to the fact that the number of χ angles is residue-dependent, which introduces variability in the side-chain conformational space that is more prominent in the unfolded ensemble.

**Figure 3:**
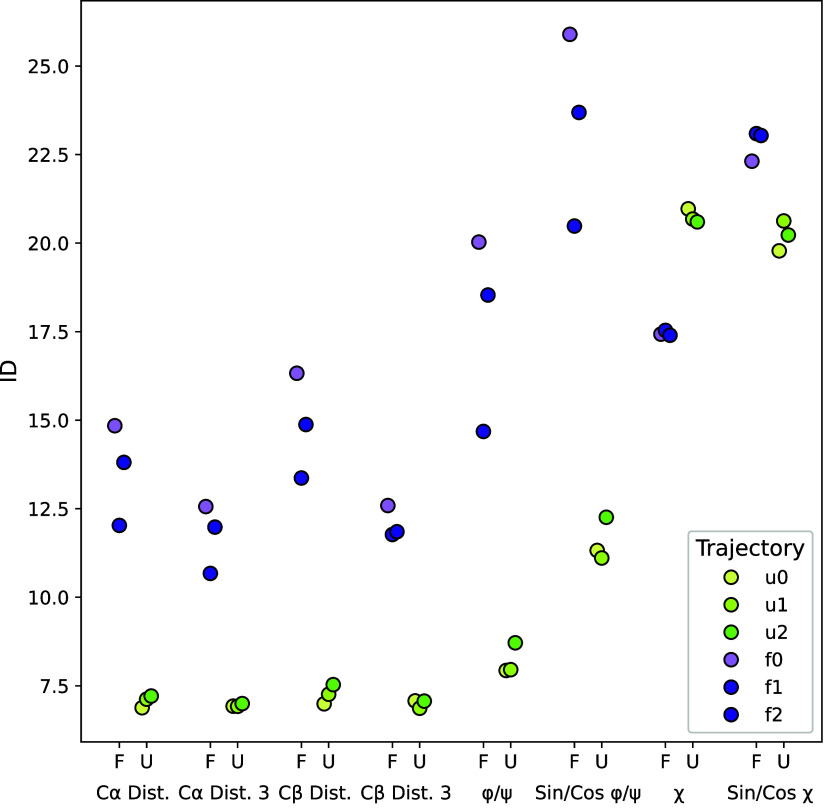
ID shifts between the folded (F) and unfolded (U) states of villin under different projections. *Dist.*: pairwise distances between all carbon–carbon pairs; *Dist. 3*: pairwise distances every third carbon; ϕ, ψ: Ramachandran angles; χ: side chain dihedrals; *Sin/Cos*: trigonometric embedding of dihedrals.

In this sense, higher ID values obtained with χ angles can be interpreted as reporting increased heterogeneity in side-chain conformational space, whereas distance- and backbone-based projections mainly track changes in tertiary contacts and backbone shape. For practical applications, we therefore recommend distance- or backbone-dihedral projections when the goal is to characterize global folding/unfolding behavior or large-scale structural transitions and χ-based projections when one is specifically interested in side-chain plasticity, for example, in binding interfaces or active sites.

#### 3.1.3 Instantaneous, Averaged, and Overall ID

As described in Section 2.2, *instantaneous, averaged,* and *overall* ID are complementary approaches that provide distinct perspectives on system behavior: instantaneous ID captures frame-by-frame variations in dimensionality (Figure 4A), whereas the time-averaged and overall ID yield compact summaries that facilitate comparison across trajectories. Although these two summary measures may appear similar, they emphasize different aspects (Supplementary Figure S1): the time-averaged ID, obtained as the mean of the instantaneous estimates, reflects the most frequently sampled conformations during the simulation. In contrast, the overall ID is computed directly from the pooled trajectory, treating it as a single dataset, and therefore quantifies the dimensionality of the conformational ensemble as a whole.

**Figure 4:**
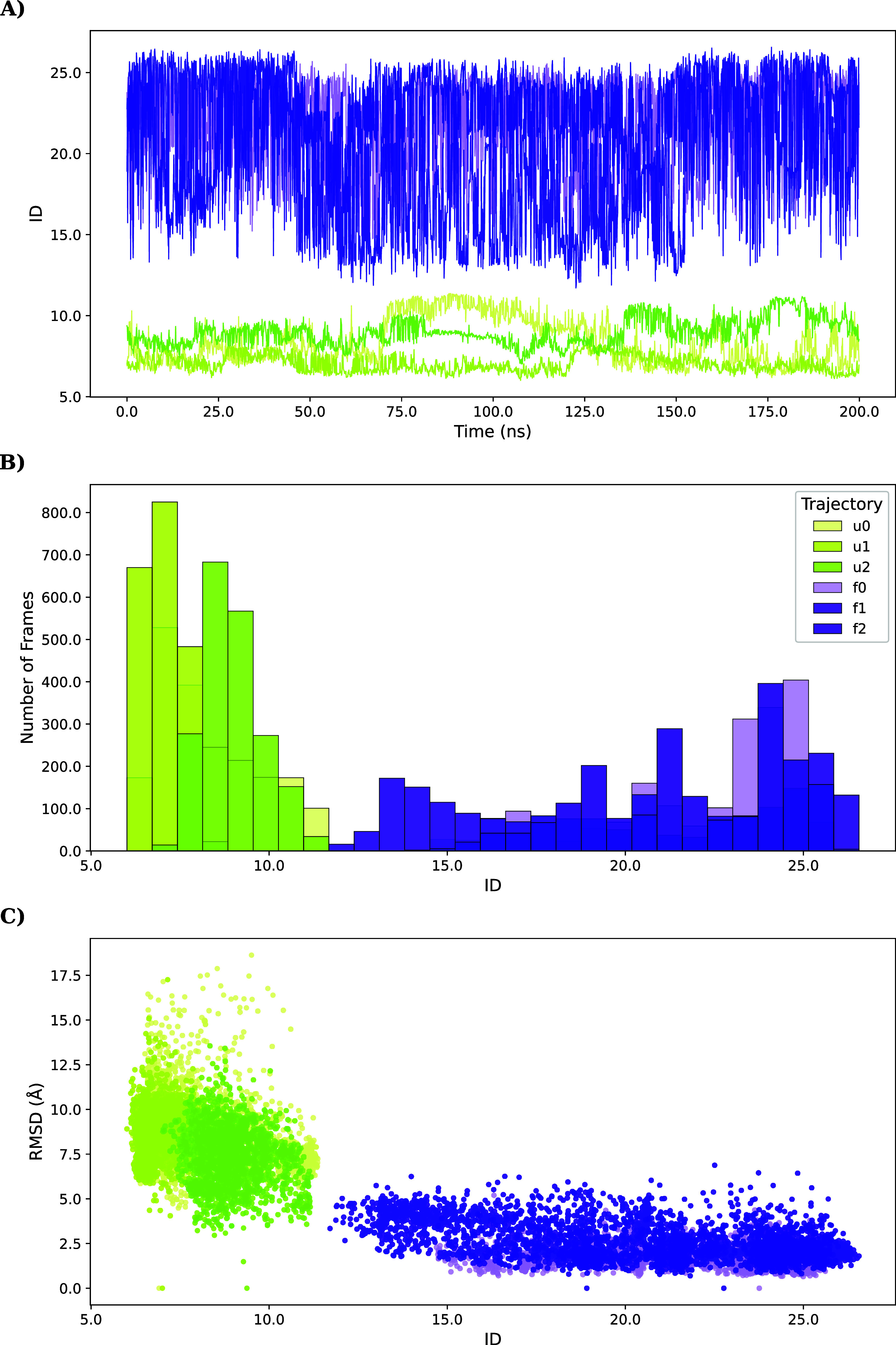
(A) Instantaneous intrinsic dimension (ID) of villin trajectories over time. (B) Distribution of ID values across the trajectory. (C) Relationship between the instantaneous ID and RMSD relative to the folded state. The separation between folded and unfolded ensembles is clearer when using ID than RMSD. States are color-coded as folded (violet) and unfolded (green). Analysis was performed on the projection to ϕ and ψ Ramachandran angles.

#### 3.1.4 Comparison with RMSD

Root-mean-square deviation (RMSD) is commonly used to quantify structural changes in proteins when a reference structure is available. Accordingly, it is natural to compare instantaneous RMSD values with the instantaneous ID values. When the RMSD is computed relative to the folded reference structure, unfolded trajectories produce higher RMSD values, whereas ID assigns higher values to the folded trajectories.

This difference reflects the nature of the two metrics: RMSD measures how much a structure deviates from a fixed reference over time, whereas ID quantifies the effective number of degrees of freedom in the motion, determined by intramolecular constraints and the types of motions available. A compact folded globule—without large flexible “unfolded hinges”—appears to explore more effective degrees of freedom. Both metrics distinguish folded from unfolded trajectories (Figure 4C), but ID provides a sharper separation, with no overlap between the distributions.

While RMSD was chosen as a widely used baseline observable, ID can also be compared with alternative order parameters, such as ID estimated from PCA’s fraction of explained variance (lPCA) or projections of the trajectory on time-lagged independent components (tICA).^16^ For both villin and NTL9, estimates based on the leading principal components (Supplementary Figures S4 and S8) or the first two time-lagged independent components (Supplementary Figures S5 and S9) yielded broader and partially overlapping distributions between folded and unfolded states. In contrast, the instantaneous ID estimated by TwoNN produced a much sharper separation with minimal overlap. These observations suggest that, at least for the systems tested here, ID captures aspects of conformational heterogeneity that are not easily summarized by linearly projected coordinates alone.

#### 3.1.5 ID by Sequence

To gain higher-resolution insight into protein dynamics, we computed sequence-local and structure-local intrinsic dimensionality (ID). In Figure 5A we show the results of section_id­() applied to a villin headpiece, partitioned into seven overlapping windows of 15 residues each, with a sliding step of three residues. Although the fixed window length and substantial overlap introduce a strong correlation between adjacent segments from the same trajectory, the ID profiles still reveal a clear distinction between folded and unfolded states, as well as minor variations among replicas within each window.

**Figure 5:**
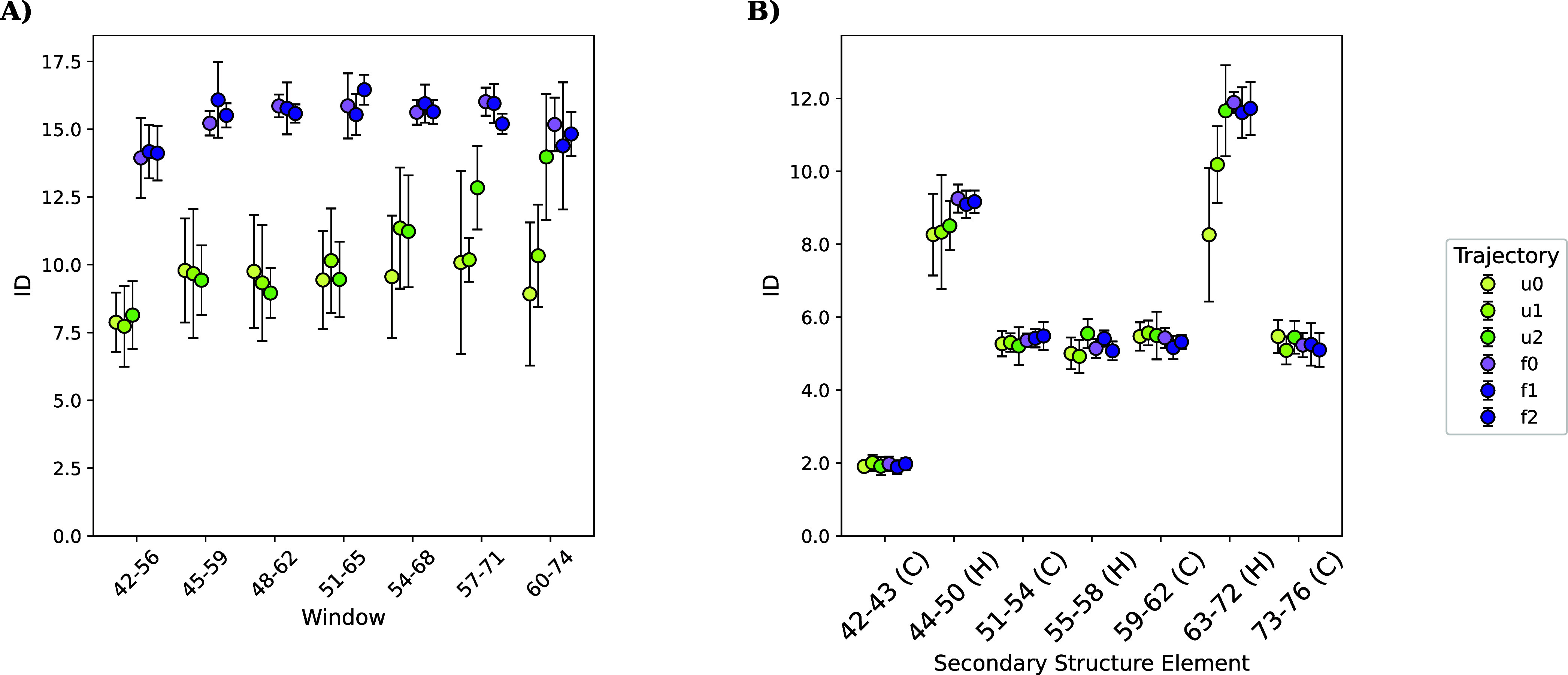
ID computed on local segments of villin. (A) Sequence-wise ID computed with section_id­() using a window of 15 and a stride of 3. (B) Secondary structure element-wise ID from secondary_structure_id­(); ranges indicate the first and last residue number of the secondary structure element (simplified DSSP: C, coil; H, helix). In both cases, ϕ and ψ dihedral angles were used as a projection.

We explored a range of window lengths and strides (5–30 residues and 1–4-residue strides) and found that the main trends distinguishing folded from unfolded segments and the location of high-ID regions were robust, while larger windows primarily smoothed out fine-scale variations in the profiles (Supplementary Figures S2 and S3).

#### 3.1.6 ID by Secondary Structure

On the other hand, the secondary_structure_id­() function subdivides the protein sequence according to the DSSP algorithm, which assigns each residue to a secondary structure element based on geometric criteria. These sections are contiguous, nonoverlapping, and variable in length, so the resulting ID values reflect both the intrinsic flexibility of each secondary structure element and a reduced influence from neighboring segments, as shown in Figure 5B, where secondary structure elements are defined on a reference folded structure (in this case, the PDB structure used as input for the topology). ID shows higher variability between sections than between folded and unfolded states, suggesting that local structural context rather than the folding state dominates the observed dimensionality.

### 3.2 Case Study: NTL9

We repeated the analysis on another protein from the same dataset, the N-terminal domain of Ribosomal Protein L9 (NTL9, PDB: 2HBA­(1–39)), a 39-residue protein carrying a single point mutation (K12M) that increases its stability by 1.9 kcal/mol.^17^ This case was chosen to validate our package’s performance on a protein with a more complex topology (Supplementary Figure S6). We selected the unbiased trajectory DESRES-Trajectory_NTL9–2-protein, evaluating its RMSD with respect to the folded structure (reference frame 12,500). Following the method described previously, we identified six segments of 200 ns each (2,000 frames), comprising three folded-state segments (f0, f1, f2) and three unfolded-state segments (u0, u1, u2) (Supplementary Table S3).

Consistent with villin results, the *TwoNN* estimator distinguishes between folded and unfolded states (Supplementary Figure S7). The projection methods tested (carbon–carbon distances and torsional angles) have a similar trend, namely, the IDs computed with χ and Ramachandran angles shift in opposite directions (Supplementary Figure S10). In absolute terms, the ID metric yielded higher values for the folded trajectories.

RMSD-versus-ID analysis followed the expected anticorrelated trend (Figure 6), with one noteworthy exception: trajectory u2 exhibited a period during which ID values were more typical of a folded segment but with a high RMSD with respect to the folded structure. This combination indicates the detection of a transient non-native, but relatively stable, folding intermediate. The intermediate, a three-helix globule (Supplementary Figure S11), can be clearly identified with a peak in the *instantaneous* ID between 160 and 180 ns (Supplementary Figure S12), but not in the *averaged* nor *overall* ID metrics (Supplementary Figure S13).

**Figure 6:**
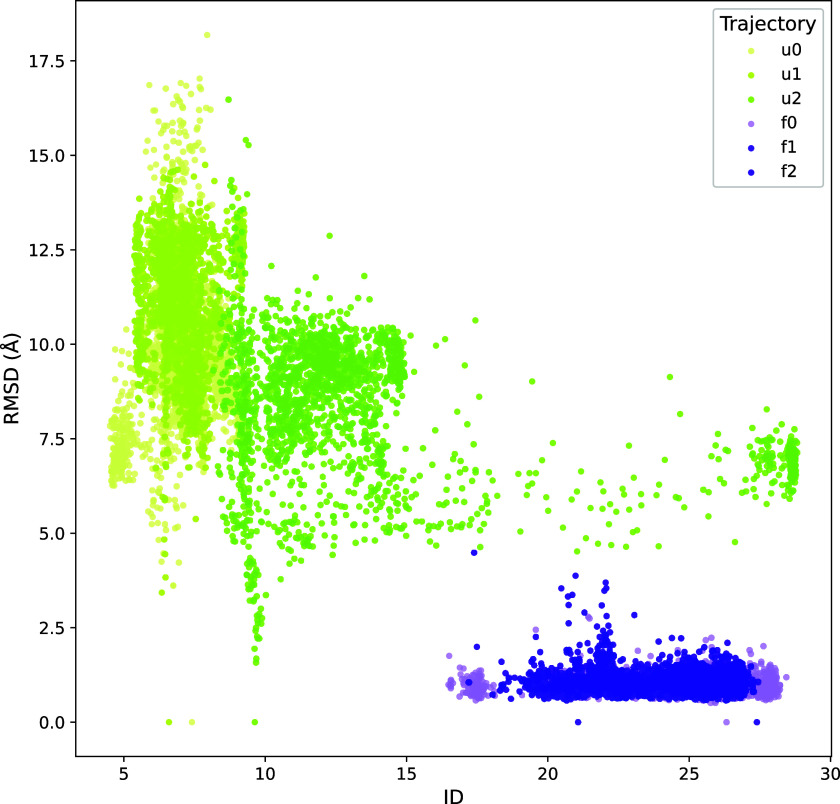
RMSD vs. instantaneous ID of NTL9 in folded (violet) and unfolded (green) states. Projection: ϕ and ψ dihedral angles.

Lastly, we evaluated the NTL9 sequence and structure locality. ID values derived from secondary_structure_id­() (Supplementary Figure S14B), predominantly influenced by the structural context, were villin-like, as expected. On the other hand, an analysis with section_id­() indicated that in trajectory u2, N-terminal windows had higher ID values, indicating an increased folded character for region 1–30 (Supplementary Figure S14A).

## 4 Discussion and Conclusions

MD simulations produce high-dimensional datasets that are often difficult to interpret directly. Estimating the ID of conformational ensembles provides a compact measure of the effective degrees of freedom and offers a complementary view of the molecular flexibility and structural heterogeneity. The package implements this idea using internal-coordinate representations and multiple estimators, enabling both trajectory-level and local region-specific analyses.

From a methodological standpoint, ID is closely related to but distinct from dimensionality estimates obtained from explicit embeddings such as PCA or tICA. Whereas these methods return an ordered set of collective coordinates and an associated explained-variance spectrum, ID summarizes in a single scalar the effective dimensionality of the manifold explored by the trajectory. A natural extension of the present work will be to systematically compare ID with the number of principal components or time-lagged independent components required to explain a given fraction (e.g., 90%) of the variance, thereby helping practitioners connect this notion to more familiar linear analysis tools.

When applied to unbiased folding–unfolding simulations, the method distinguishes folded, unfolded, and transiently folded states. These results show that intrinsic dimensionality reflects changes in dynamic heterogeneity and flexibility, thereby complementing established order parameters used in folding studies.^18,19^

We remark that an extended structure spends most of its time moving along a few soft collective directions (such as overall expansion and compaction), whereas a relatively compact globule supports many more, albeit smaller-amplitude, fluctuation modes. This reconciles the (apparently paradoxical) higher ID of the folded state with the intuitive picture that an unfolded chain has more freedom, as seen in Section 3 in the case studies of villin and NTL9.

More generally, estimating ID highlights collective dynamical regimes that may be obscured in the full coordinate space and may inform the construction of data-driven collective variables and features for Markov state modeling.^4,20,21^

The package’s modular design, open-source availability, and compatibility with existing MD analysis workflows make it broadly applicable to diverse biomolecular systems, from proteins to nucleic acids and complexes. By bridging concepts from nonlinear data analysis and biophysical modeling, MDIntrinsicDimension hopefully offers a novel lens for exploring molecular flexibility and conformational landscapes.

## Supplementary Material



## Data Availability

The code is freely available from the GitHub repository giorginolab/MDIntrinsicDimension, together with extensive documentation and self-contained notebooks, which reproduce figures and results of this paper. The repository also includes regression tests and continuous integration workflows that test the analysis functions to ensure stability as features and dependencies evolve. Data for the case studies is available from ref 11.

## Notes

List of methods and their abbreviations, frame ranges used in the analysis, extended plots, and analysis for villin HP35 and NTL9 trajectories (PDF)
