# Supplementary material for: MDIntrinsicDimension: Dimensionality-Based Analysis of Collective Motions in Macromolecules from Molecular Dynamics Trajectories
Source: J Chem Inf Model. 2026 Feb 26;66(5):2443–50. doi: 10.1021/acs.jcim.5c02716 (PMC12977056; doi:10.1021/acs.jcim.5c02716)
Supplement: Supplementary file 1 [file ci5c02716_si_001.pdf]

Supporting Information

MDIntrinsicDimension: Dimensionality-Based Analysis of Collective Motions in Macromolecules from Molecular Dynamics Trajectories

Irene Cazzaniga[1], Toni Giorgino[1]

[1]Istituto di Biofisica (IBF-CNR), Consiglio Nazionale delle Ricerche, Milano, Italy.

This file contains the list of methods and their abbreviations, frame ranges used in the analysis, extended plots for villin HP35 and NTL9. The two-nearest neighbours (TwoNN) estimator was used for all plots unless indicated otherwise.

1 Supporting Information for the Methods Section

**Estimator name**	**Abbreviation**	**Type**	**Reference**
Correlation (fractal) dimensionality	CorrInt	local	[1]
Dimensionality from angle and norm concentration	DANCo	global	[2]
Expected simplex skewness	ESS	global	[3]
Fisher separability	FisherS	global	[4]
Weighted average kNN distances	KNN	local	[5]
Local principal component analysis	lPCA	local	[6]
Manifold-adaptive fractal dimension	MADA	local	[7]
Minimum neighbor distance–maximum likelihood	MiND_ML	global	[8]
Maximum likelihood estimator	MLE	local	[9]
Method of moments	MOM	local	[10]
Tight localities estimator	TLE	global	[11]
Two-nearest neighbours	TwoNN	global	[12]

Table S1: ID estimators supported by the `scikit-dimension` library (alphabetically ordered).

2 Supporting Information for the villin HP35 Case Study

TS	Key	Frames	State
000	u0	0–2000	Unfolded
001	f0	0–2000	Folded
001	u1	5000–7000	Unfolded
004	f1	8000–10000	Folded
005	u2	1700–3700	Unfolded
005	f2	5000–7000	Folded

Table S2: Subsections of the villin trajectories selected for analyses, split into three periods in the folded state and three in the unfolded state. *TS* is the trajectory segment, i.e. the dotted part in trajectory file names `2F4K-protein-0-....dcd` from [13]; *Key* is the corresponding abbreviation used in the legends.

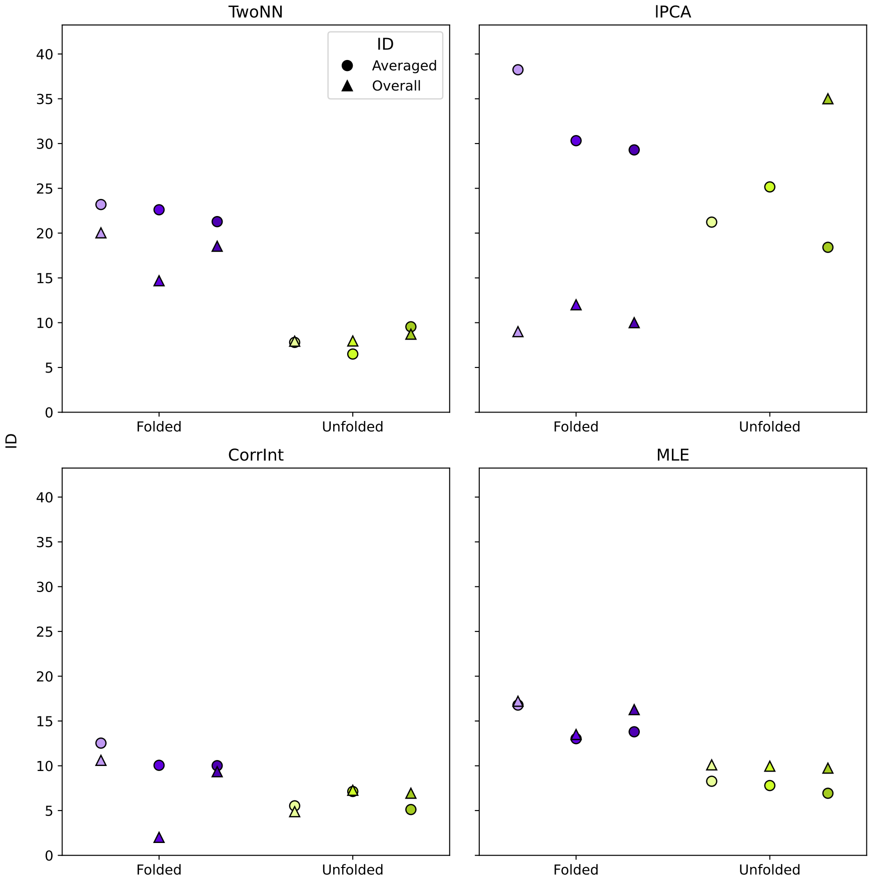

Figure S1: Averaged (circles) and overall (triangles) ID of the villin trajectories. The two summary metrics take on similar values for most estimators (only four are shown here for clarity). lPCA is an exception but, as discussed in the main text, it does not discriminate states clearly. In all cases, ϕ and ψ dihedral angles were used as a projection.

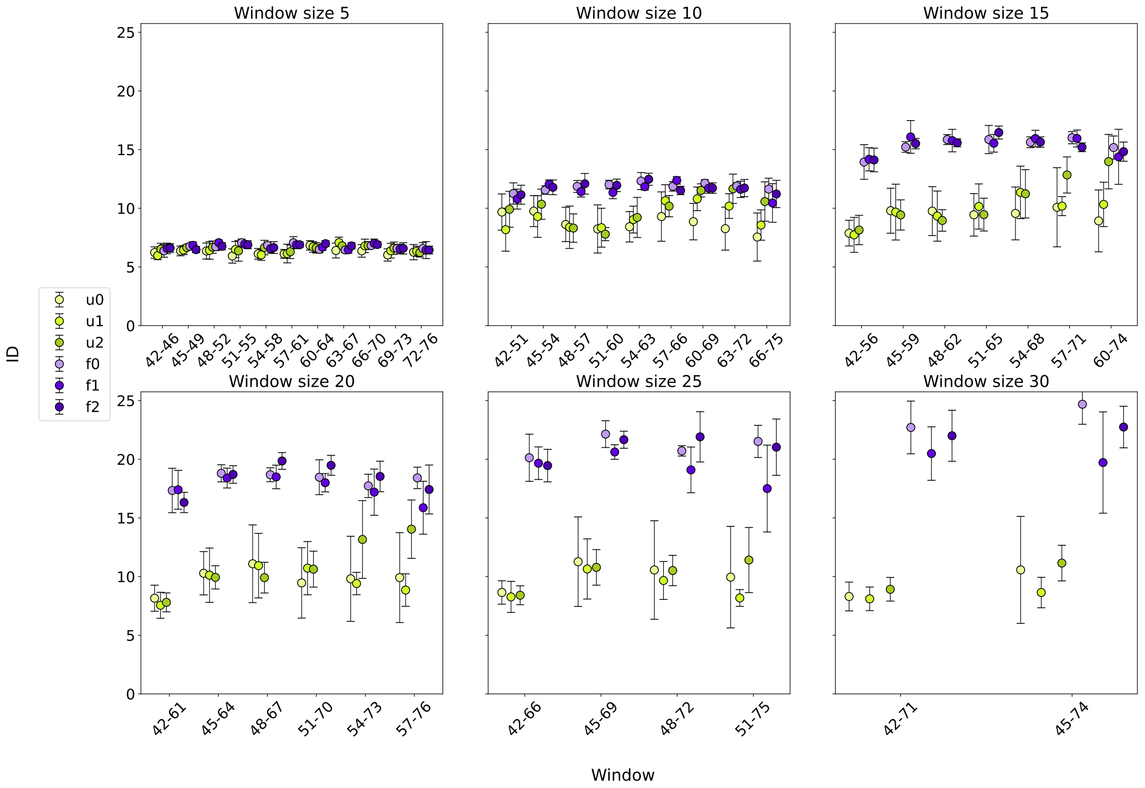

Figure S2: Sensitivity of section-wise ID (`section_id()`) to the window size. The window size strongly affects the ID, especially in case of folded states: for very small windows (e.g. 5) ID saturates and does not detect structural differences between the folded or unfolded states; at larger window sizes the classification becomes clearly distinct. Even larger window sizes blur sequence details to the point where the ID approaches the whole-protein value (e.g. window size of 30, with 35 total residues).

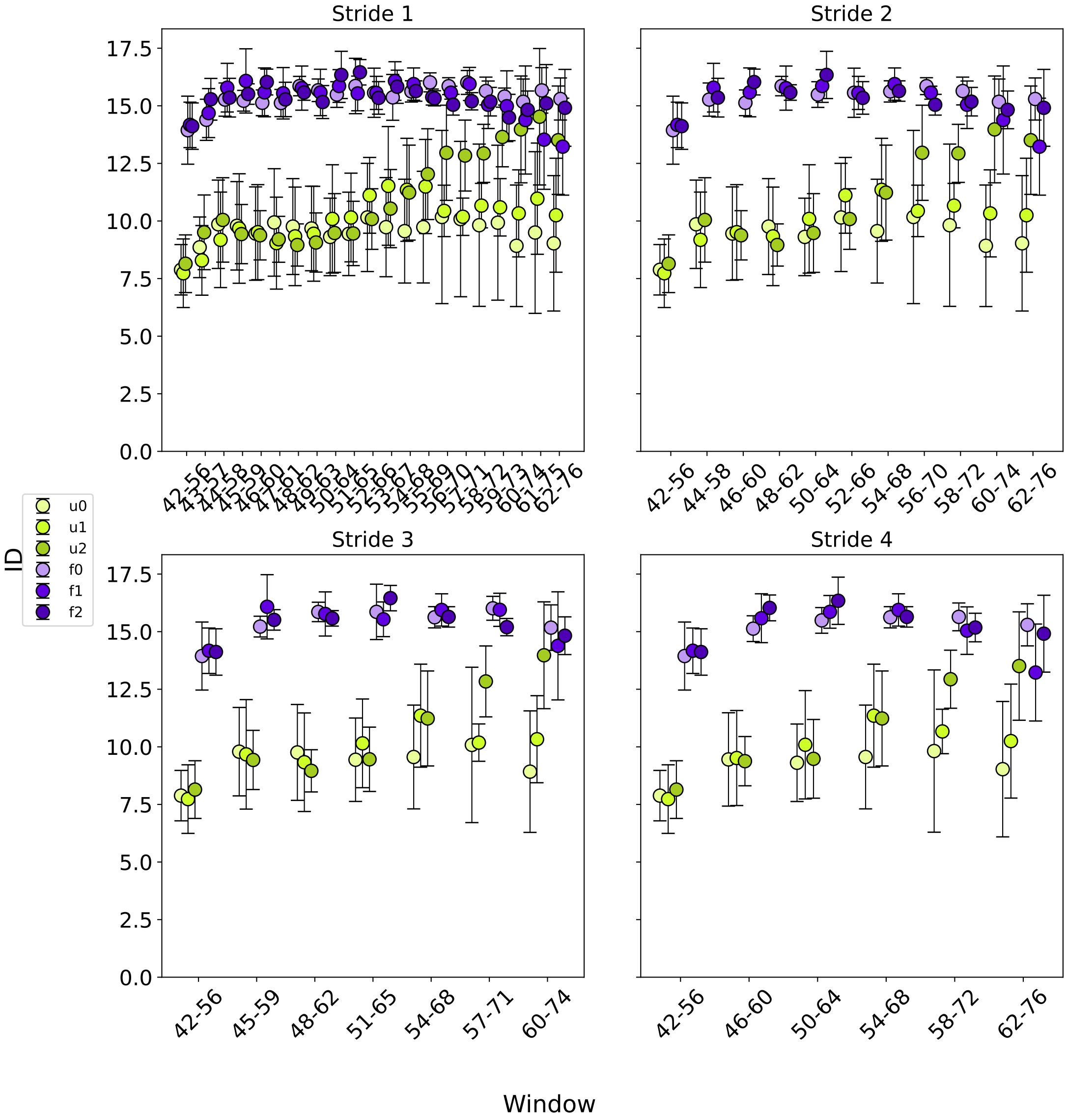

Figure S3: Sensitivity of section-wise ID (`section_id()`) to the stride. Expectedly, a large stride makes the ID profiles sparser without altering their trend.

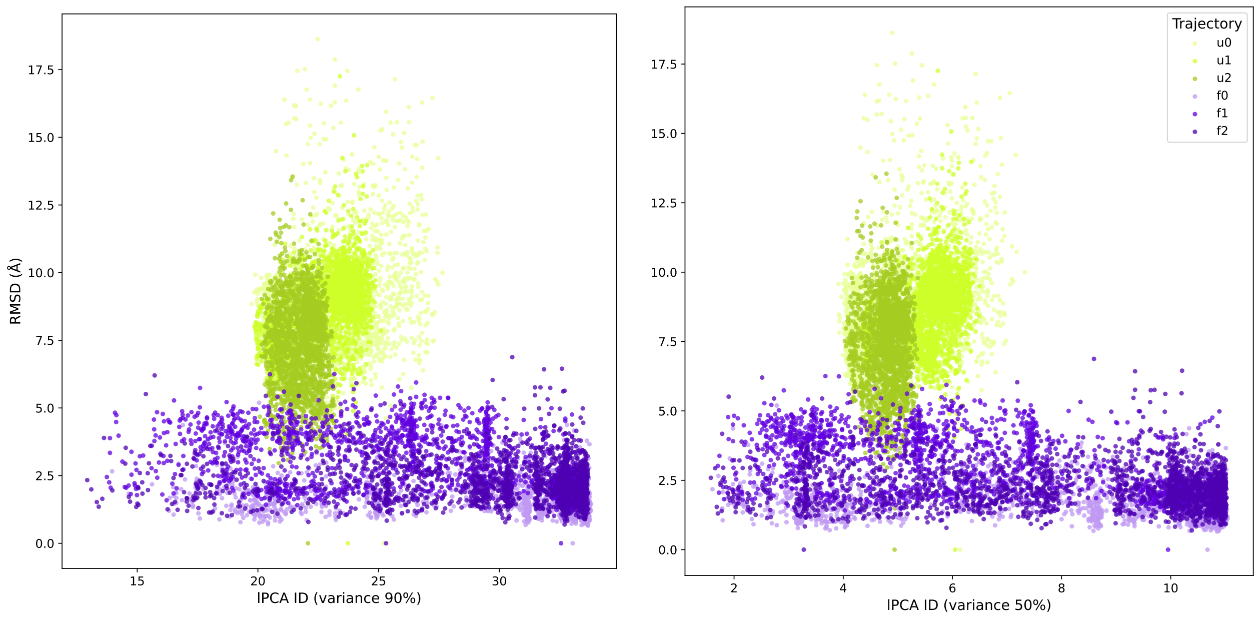

Figure S4: Comparison between PCA-based ID estimation and RMSD on MD simulations of villin. The horizontal axis corresponds to the instantaneous ID estimated as the number of eigenvalues accounting for 90% (left) or 50% (right) of the local variance. Both thresholds fail to separate the folded (violet) and unfolded (green) states trajectories. Hence, ID computed via modern, nonlinear estimators like TwoNN provide a more precise estimate. Projection: ϕ and ψ dihedral angles.

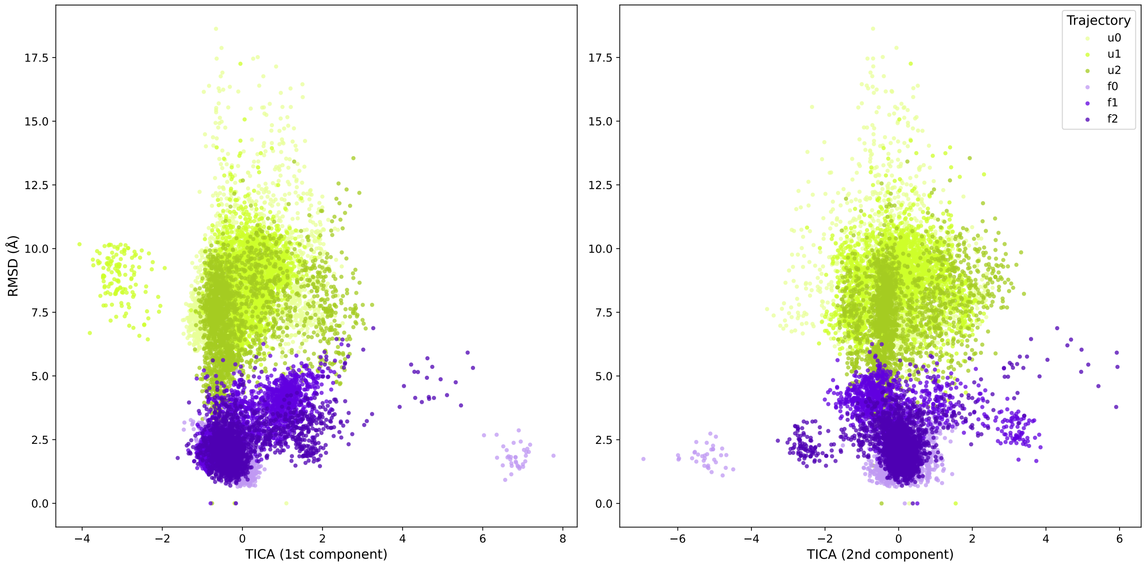

Figure S5: Comparison between tICA (first and second components) and RMSD on MD simulations of villin. Neither of the two tICA components discriminates the folded (violet) vs. unfolded (green) trajectories. Projection: ϕ and ψ dihedral angles.

3 Supporting Information for the NTL9 Case Study

In the following, "NTL9" refers to the N-terminal Domain of Ribosomal Protein L9 (NTL9, PDB:2HBA(1-39)). Trajectories refer to `DESRES-Trajectory NTL9-2-protein` dataset from the D. E. Shaw Research fast-folding proteins [13]. If not stated otherwise, the estimator of choice is *TwoNN* [12], and the projections are computed from ϕ and ψ Ramachandran angles.

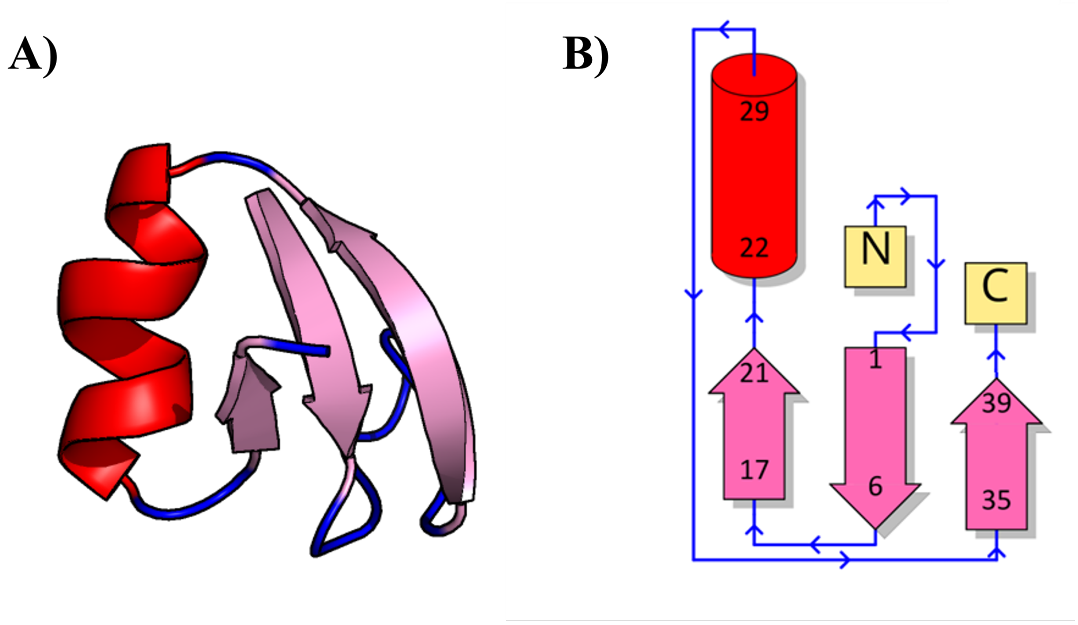

Figure S6: NTL9 (PDB 2HBA), 39-residue protein structure (A), colored by secondary structure and corresponding topology diagram (B).

Traj	Key	Frames	State
000	u0	0–2000	Unfolded
050	f0	0–2000	Folded
080	u1	0–2000	Unfolded
100	f1	0–2000	Folded
124	u2	0–2000	Unfolded
194	f2	0–2000	Folded

Table S3: Subsections of the NTL9 trajectories selected for analyses, sliced into three periods in the folded state and three in the unfolded state. "Traj" correspond the trailing part of data files names `NTL9-2-protein-....dcd` and accompanying paper [13]; "Key" is the corresponding abbreviation used in the legends.

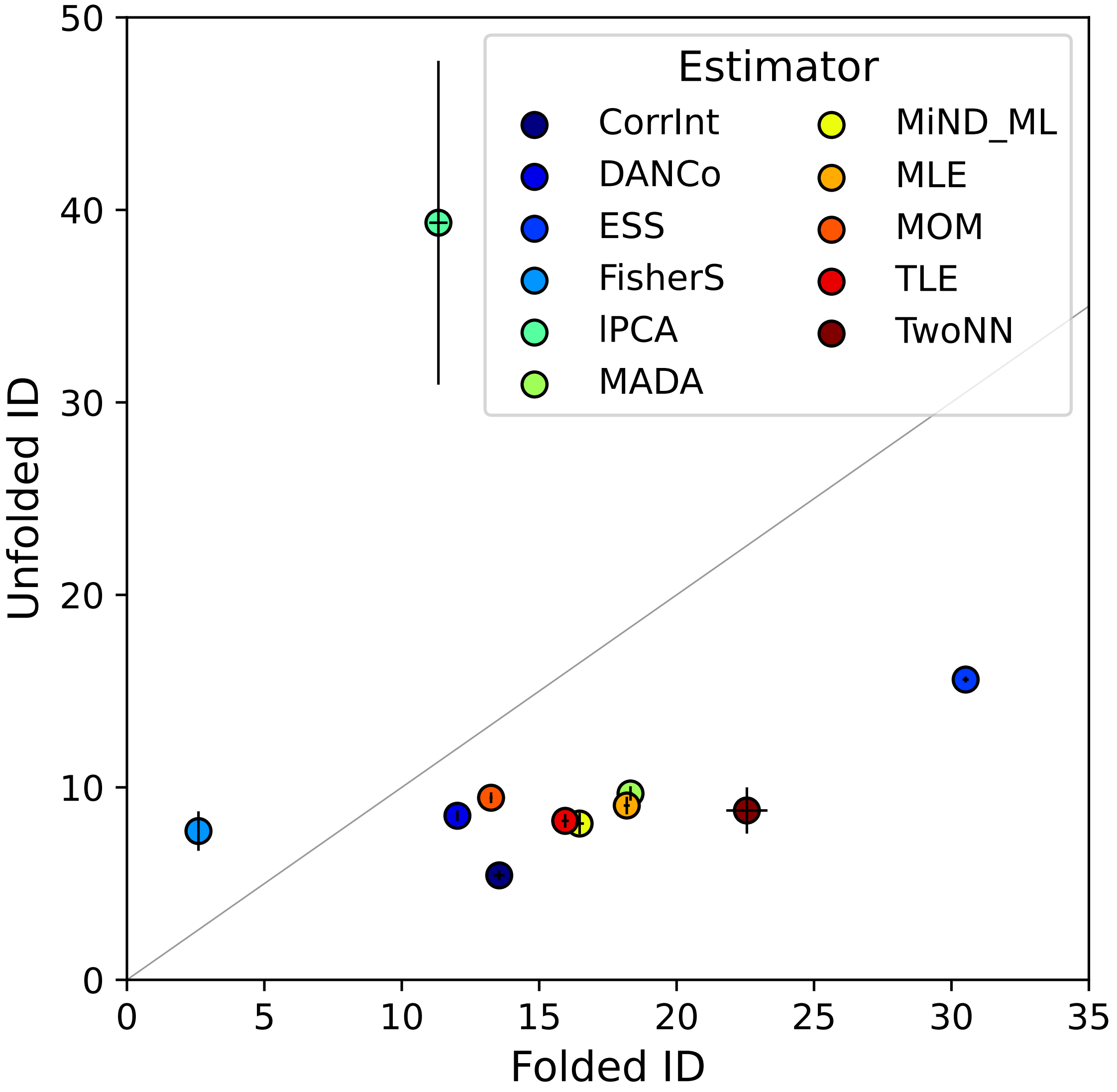

Figure S7: Folded–vs–unfolded ID of the NTL9 dynamic manifold, computed bu the estimators available at `scikit-dimension` package. Each point represents the mean value of the folded states (x axis) and unfolded states (y axis). Error bars parallel to each axis indicate the standard deviation from the mean for the respective state.

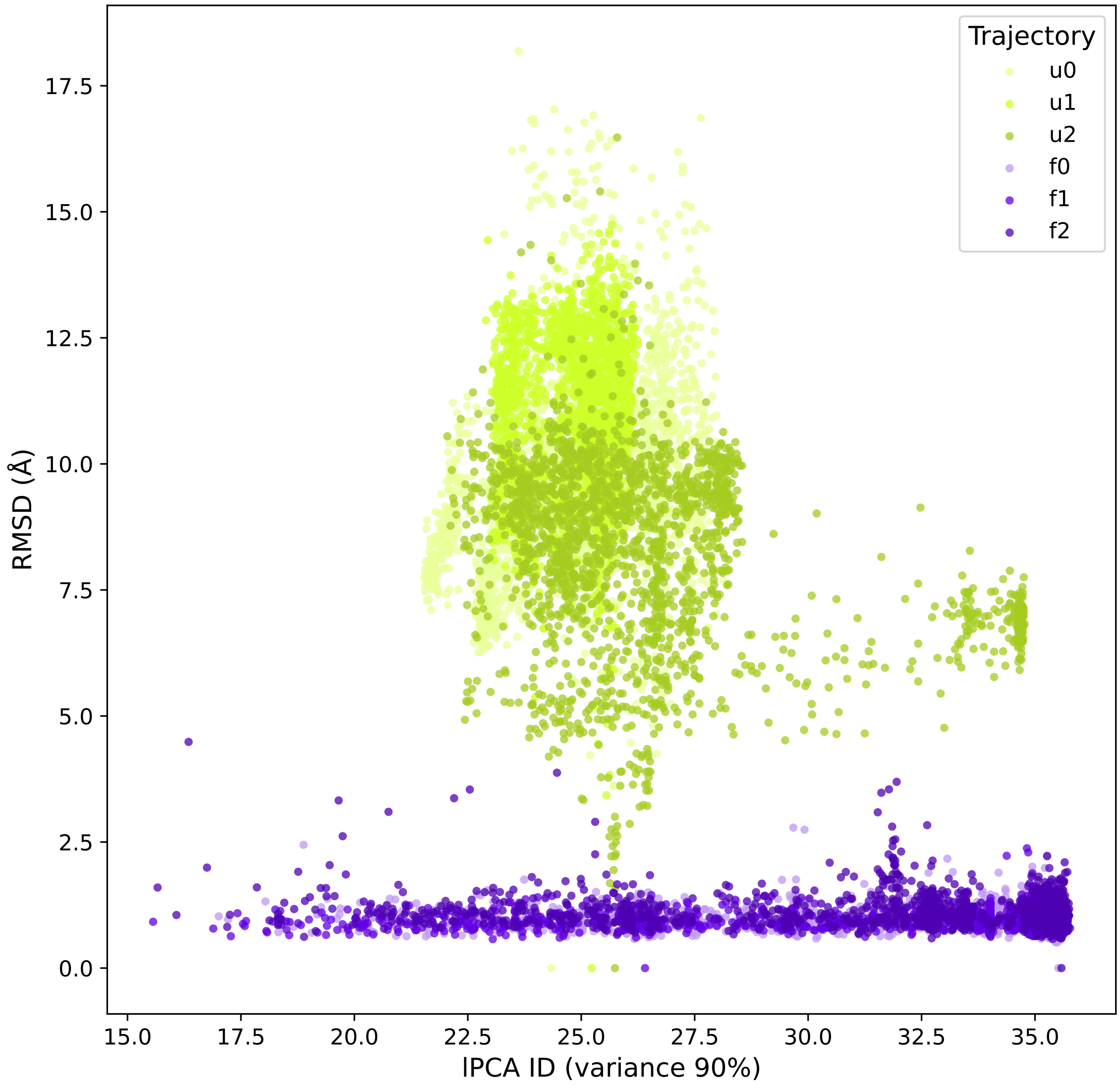

Figure S8: RMSD versus lPCA of NTL9 in folded (violet) and unfolded (green) states. Projection: ϕ and ψ dihedral angles. Refer to caption of Supplementary Figure S4 (left) for details. Similarly to villin, lPCA does not discriminate the folding state.

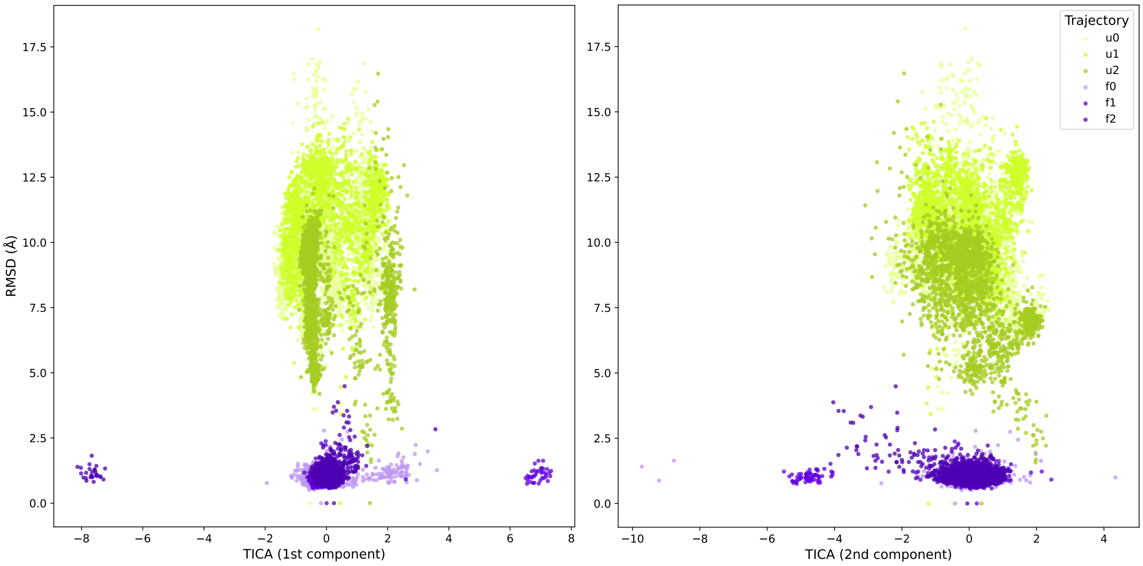

Figure S9: RMSD versus tICA components 1 and 2 of NTL9 in folded (violet) and unfolded (green) states. Projection: ϕ and ψ dihedral angles. Refer to caption of Supplementary Figure S5 for details.

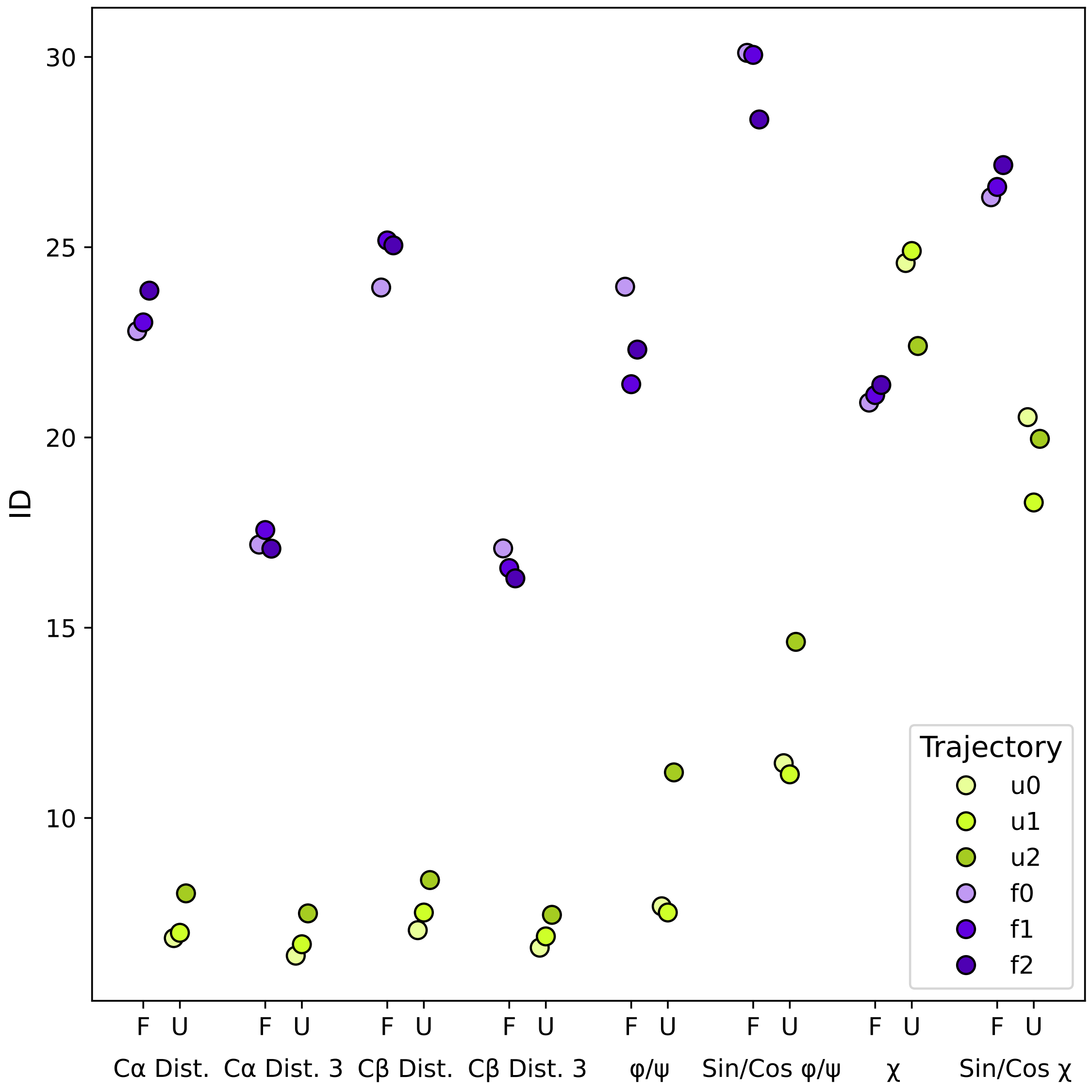

Figure S10: Differences in ID between folded (f) and unfolded (u) states on NTL9 under different projections. *Dist.*: pairwise distances between all carbon–carbon pairs; *Dist. 3*: pairwise distances every 3rd carbon; ϕ, ψ: Ramachandran angles; χ: sidechain dihedrals; *Sin/Cos*: trigonometric embedding of angles.

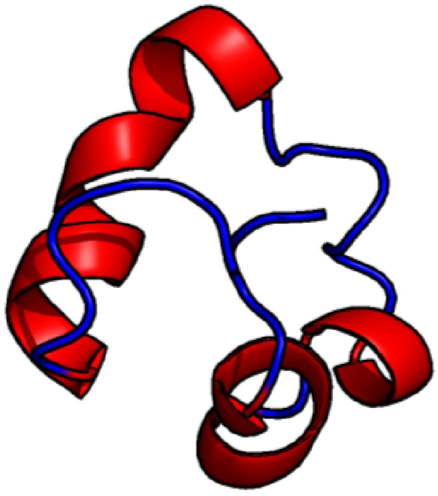

Figure S11: Transiently folding intermediate of NTL9 found in trajectory `u2` at frame 1700.

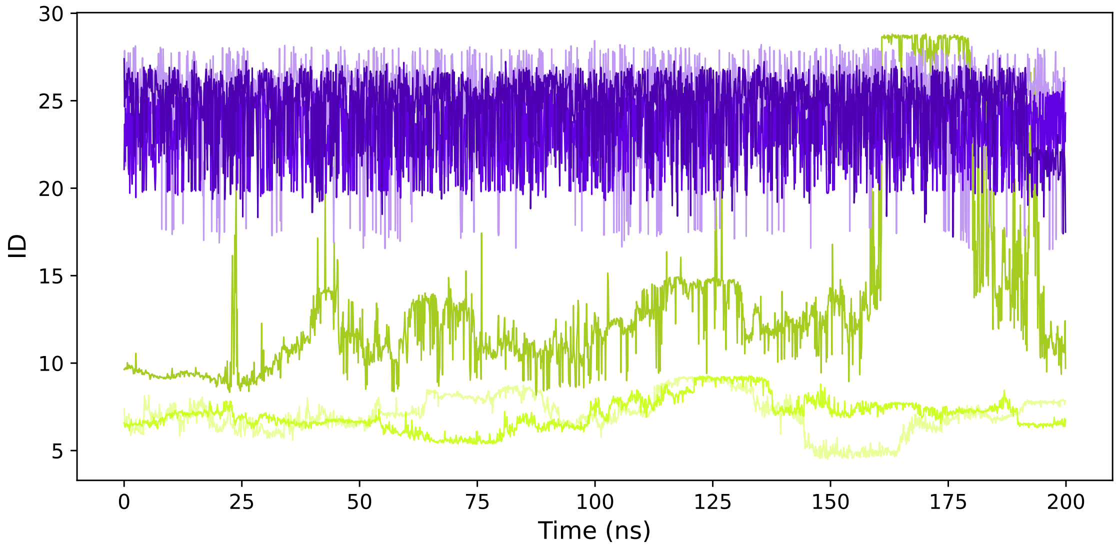

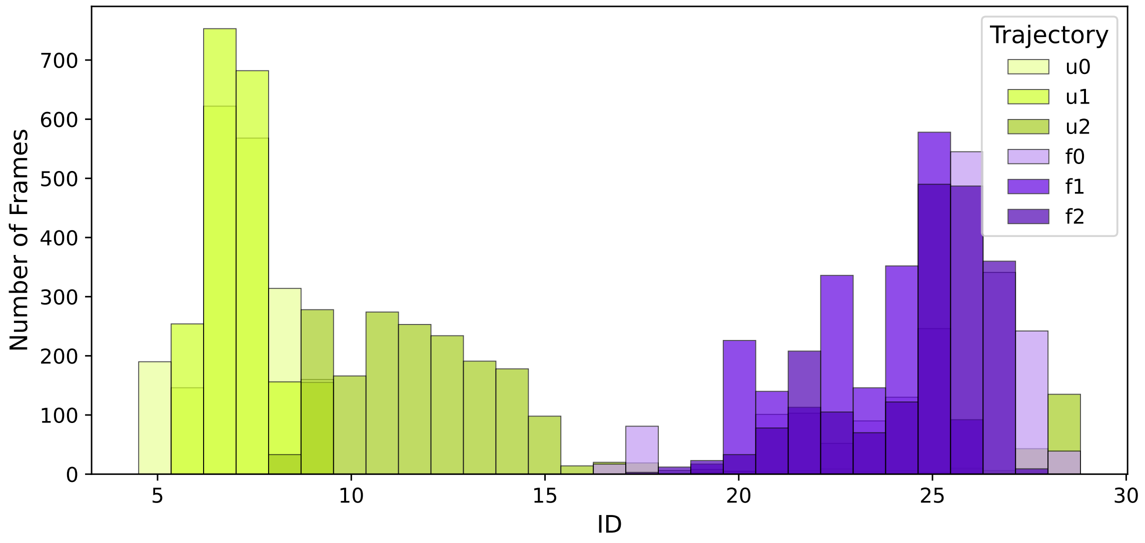

Figure S12: Instantaneous ID over time (top panel) and ID distribution frequency (bottom panel) along the trajectory. In violet, folded protein trajectories; in green, unfolded ones.

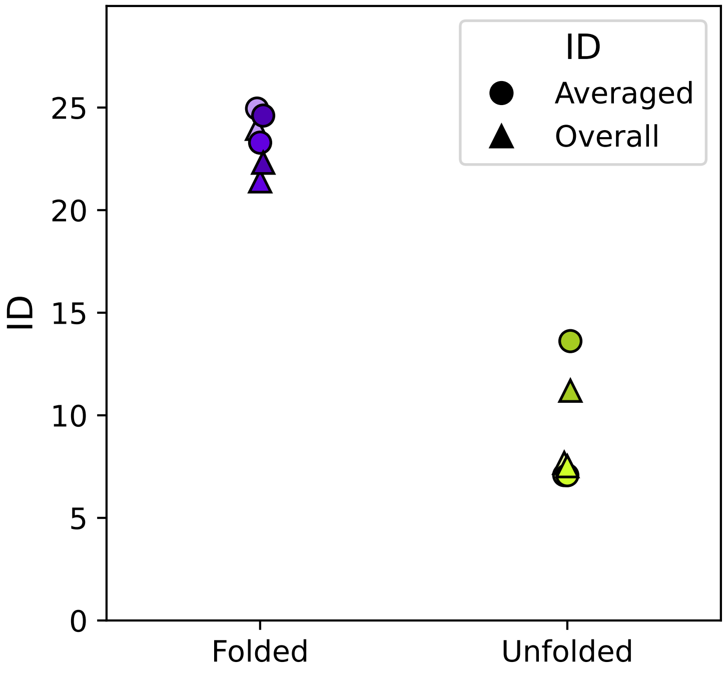

Figure S13: Averaged (circles) and overall (triangles) ID from individual trajectories.

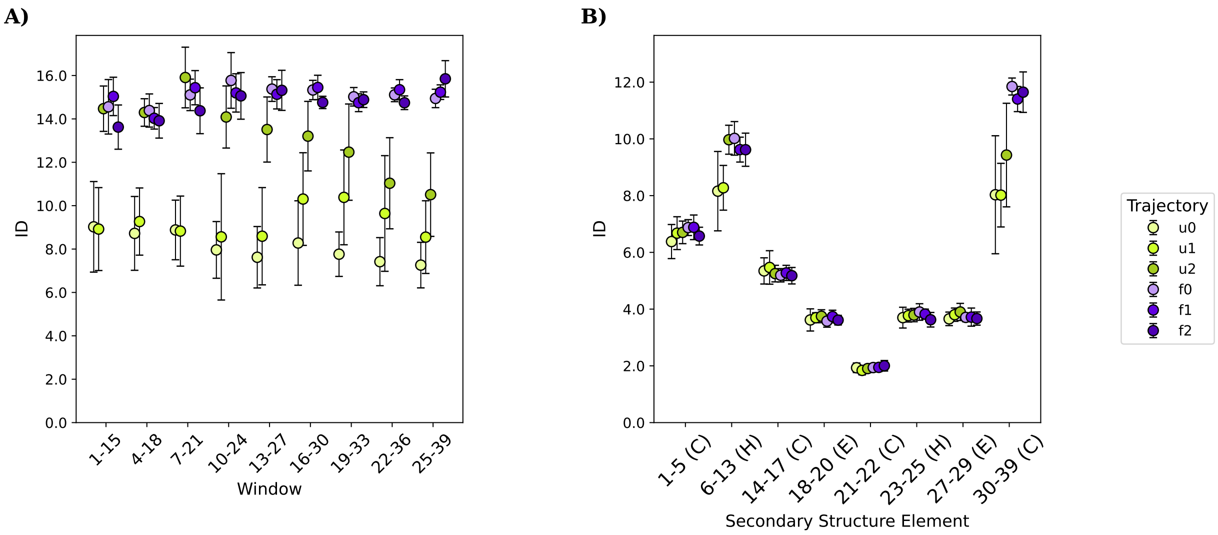

Figure S14: ID computed locally and on structural regions: (A) Sequence-wise ID computed with `section_id()` using a *window* of 15 and *stride* of 3; (B) Structure-wise ID computed with `secondary_structure_id()`, based on simplified 3-class DSSP assignments.

References

[1] Grassberger P, Procaccia I. Measuring the strangeness of strange attractors. Physica D: Nonlinear Phenomena. 1983 Oct;9(1-2):189-208. Available from: https://linkinghub.elsevier.com/retrieve/pii/0167278983902981.

[2] Ceruti C, Bassis S, Rozza A, Lombardi G, Casiraghi E, Campadelli P. DANCo: An intrinsic dimensionality estimator exploiting angle and norm concentration. Pattern Recognition. 2014 Aug;47(8):2569-81. Available from: https://linkinghub.elsevier.com/retrieve/pii/S003132031400065X.

[3] Johnsson K, Soneson C, Fontes M. Low Bias Local Intrinsic Dimension Estimation from Expected Simplex Skewness. IEEE Transactions on Pattern Analysis and Machine Intelligence. 2015 Jan;37(1):196-202. Available from: http://ieeexplore.ieee.org/document/6866171/.

[4] Albergante L, Bac J, Zinovyev A. Estimating the effective dimension of large biological datasets using Fisher separability analysis. In: 2019 International Joint Conference on Neural Networks (IJCNN). Budapest, Hungary: IEEE; 2019. p. 1-8. Available from: https://ieeexplore.ieee.org/document/8852450/.

[5] Carter KM, Raich R, Hero AO. On Local Intrinsic Dimension Estimation and Its Applications. IEEE Transactions on Signal Processing. 2010 Feb;58(2):650-63. Available from: http://ieeexplore.ieee.org/document/5233815/.

[6] Fukunaga K, Olsen DR. An Algorithm for Finding Intrinsic Dimensionality of Data. IEEE Transactions on Computers. 1971 Feb;C-20(2):176-83. Conference Name: IEEE Transactions on Computers.

[7] Farahmand AM, Szepesvári C, Audibert JY. Manifold-adaptive dimension estimation. In: Proceedings of the 24th international conference on Machine learning. Corvalis Oregon USA: ACM; 2007. p. 265-72. Available from: https://dl.acm.org/doi/10.1145/1273496.1273530.

[8] Rozza A, Lombardi G, Ceruti C, Casiraghi E, Campadelli P. Novel high intrinsic dimensionality estimators. Machine Learning. 2012 Oct;89(1-2):37-65. Available from: http://link.springer.com/10.1007/s10994-012-5294-7.

[9] Levina E, Bickel PJ. Maximum Likelihood Estimation of Intrinsic Dimension. In: Saul LK, Weiss Y, Bottou L, editors. Advances in Neural Information Processing Systems 17. MIT Press; 2005. p. 777-84. Available from: http://papers.nips.cc/paper/2577-maximum-likelihood-estimation-of-intrinsic-dimension.pdf.

[10] Amsaleg L, Chelly O, Furon T, Girard S, Houle ME, Kawarabayashi Ki, et al. Extreme-value-theoretic estimation of local intrinsic dimensionality. Data Mining and Knowledge Discovery. 2018 Nov;32(6):1768-805. Available from: http://link.springer.com/10.1007/s10618-018-0578-6.

[11] Amsaleg L, Chelly O, Houle ME, Kawarabayashi Ki, Radovanović M, Treeratanajaru W. Intrinsic Dimensionality Estimation within Tight Localities: A Theoretical and Experimental Analysis. arXiv; 2022. ArXiv:2209.14475 [cs]. Available from: http://arxiv.org/abs/2209.14475.

[12] Facco E, D'Errico M, Rodriguez A, Laio A. Estimating the intrinsic dimension of datasets by a minimal neighborhood information. Scientific Reports. 2017 Sep;7(1):12140. Available from: https://www.nature.com/articles/s41598-017-11873-y.

[13] Lindorff-Larsen K, Piana S, Dror RO, Shaw DE. How Fast-Folding Proteins Fold. Science. 2011 Oct;334(6055):517-20. Available from: https://www.science.org/doi/10.1126/science.1208351.
